# Effect of low power laser in biomodulation of cultured osteoblastic cells of Wistar rats^1^

**DOI:** 10.1590/s0102-8650201900210

**Published:** 2019-02-28

**Authors:** Maria Jose Misael da Silva Morsoleto, Valeria Sella, Paula Machado, Fernando do Bomfim, Maria Helena Fernandes, Fernando Morgado, Gaspar de Jesus Lopes, Helio Plapler

**Affiliations:** I Postdoctoral, Postgraduate Program in Interdisciplinary Surgical Sciences, Universidade Federal de São Paulo (UNIFESP), Brazil. Design, intellectual and scientific content of the study; acquisition and interpretation of data; manuscript preparation and writing.; II Fellow PhD degree, Postgraduate Program in Interdisciplinary Surgical Science, UNIFESP, Sao Paulo-SP, Brazil. Conception and design of the study.; III Physiotherapist, Postgraduate Program in Interdisciplinary Surgical Sciences, UNIFESP, Sao Paulo-SP, Brazil. Technical procedures.; IV Fellow PhD degree, Postgraduate Program in Interdisciplinary Surgical Sciences, UNIFESP, Sao Paulo-SP, Brazil. Technical procedures.; V Associate Professor, Department of Pharmacology and Cellular Compatibility, Dental Medicine Faculty, Universidade do Porto, Portugal. Histopathological examinations, Analysis and interpretation of data.; VI Associate Professor, Department of Biology, Universidade de Aveiro, Portugal. Analysis and interpretation of data, statistics analysis.; VII Associate Professor, Department of Surgery, Medical School, UNIFESP, Sao Paulo-SP, Brazil. Critical revision, final approval.; VIII Associate Professor, Department of Surgery, Medical School, UNIFESP, Sao Paulo-SP, Brazil. Conception, design, intellectual and scientific content of the study; critical revision.

**Keywords:** Cells, Cultured, Cell Proliferation, Low-Level Light Therapy, Rats

## Abstract

**Purpose:**

To analyze aspects of the biomodulating effect of light in biological tissues, bone cells from surgical explants of the femur of rats were irradiated with low intensity laser.

**Methods:**

Bone cells were cultured and irradiated with LASER light (GaAlAs). Growth, cell viability, mineralized matrix formation, total protein dosage, immunostimulatory properties, cytochemical analysis, gene expression of bone proteins were examined using live cell imaging and cell counting by colorimetric assay. The gene expression of: alkaline phosphatase (ALP), type 1 collagen, osteocalcin and osteopontin through the real-time polymerase chain reaction.

**Results:**

At 8 days, the viability of the irradiated culture was 82.3% and 72.4% in non-irradiated cells. At 18 days, the cellular viability (with laser) was 77.42% and 47.62% without laser. At 8 days, the total protein concentration was 21.622 mg / mol in the irradiated group and 16, 604 mg / mol in the non-irradiated group and at 18 days the concentration was 37.25 mg / mol in the irradiated group and 24, 95 mg / mol in the non-irradiated group.

**Conclusion:**

The laser interfered in the histochemical reaction, cell viability, matrix mineralization, and maintained the cellular expression of proteins

## Introduction

 The low intensity laser emits non-ionizing electromagnetic radiation with a power of less than one Watt, where the temperature rise does not exceed 1ºC, without destructive potential with p hotochemical and photoelectric action. It presents a cumulative effect of the dose on the target tissue, triggering analgesic, anti-inflammatory and biostimulation action^1^. In the application of this type of laser, we irradiate the target cell with low energy density, but enough to stimulate its membranes and organelles, inducing irradiated cells to biomodulation, establishing a state of normality in the irradiated region. This type of laser is called “therapeutic laser” Research has shown that the energy emitted by the laser is absorbed by intracellular chromophores and converted into metabolic energy; this cellular photostimulation is attributed to the activity of the respiratory chain of mitochondria. When the biological tissue absorbs light, a transfer of energy occurs. The energy emitted by the laser is absorbed by intracellular chromophores and converted into metabolic energy promoting cellular signaling and increasing the synthesis of ATP, RNA and proteins^2^.

 Recent studies have shown that the low intensity pulsed laser applied to osteoblast cultures from rat calvaria promotes proliferation, differentiation and changes the RANKL/OPG ratio, besides inhibiting osteoclastic differentiation, increasing adhesion, proliferation and differentiation. In human bone, increased cell viability and proliferation, alkaline phosphatase activity (ALP), and the expression of osteopontin and sialoprotein^3^

 Osteoblastic cells are stimulated by low intensity laser radiation to modify the production of mediators as a function of time, frequency and dose applied, mainly in laser with wavelength in the range of 635nm and 805nm^4^.

 Knowledge of the effect of laser radiation on certain cell types alone may provide important information about the interaction of light in the body as a whole1,4, specifically when irradiating a cell culture from a surgical explant into femoral flaps of wistar rats. Monitoring each step of cellular development when subjected to low-power laser radiation^5^.

 The technique of explant cultivation has advantages because it allows the reduced use in the number of animals used in laboratory experiments, since numerous explants are produced from a single donor piece^6^, allowing even greater environmental control for in vitro experimentation when compared with the model in vivo. Associated with these advantages, one must consider the bioethical question. Today’s society increasingly demands control over the use of experimental animals. In this sense, the explant cultivation model meets, in addition to the ethical requirements, also to the legal ones. The National Health Council standardization (Decree 93.933 approved in 1987) establishes that every study must be planned in such a way as to obtain the maximum information using the least number of animals^7^.

 The cell culture model using surgical explants is a technique still in improvement, but effective in laboratory research^8^. These models allow preserving important cell-cell and cell-matrix interactions in order to better understand the behavior of cells in their natural three-dimensional environment. Thus, the use of ex vivo bone in explant cultures may often be of greater physiological relevance than the use of bi-dimensional primary cells cultured in vitro. These models can be used to analyze the behavior and interactions between cells in bone repair or induced cancer^6^.

 Human and rodent cell cultures have contributed to the elucidation of pathophysiological aspects of bone metabolism and the possibility of modulating them with drugs, metals, light, pressure and temperature, among other elements. The systems used include primary cultures of bone cells obtained from trabecular bone, bone marrow cultures, various cell lines obtained from normal bone tissue and also tumor cell lines^8^. Each model has its own limitations. Bone cell cultures must have morphological and functional parameters characteristic of the phenotype of the cells to be studied. The expression of a particular cell phenotype in culture depends fundamentally on the biological material used, on how it is handled and on the culture conditions, especially the culture medium, on the addition of substances that interfere with proliferation, differentiation and culture time. Advantages including the use of cell cultures are: control of environmental conditions, independent analysis of parameters, high number of tests in a short timeframe, reduction in the use of animal tests and less expensive experimental techniques than the animal experimentation. The main disadvantages include: loss of phenotypic characteristics, artificial biological system and absence of important signs^9^ ^-,16^.

## Methods

 This project was approved by the Research Ethics Committee, Universidade Federal de São Paulo (No. 118/2010).

### Osteoblast cell culture

 The cells of the osteoblastic lineage were obtained from bone fragments, 2 mm long, from the surgical explants of femur fractures. Cells were isolated and cultured as described by Beloti^10^ and Binderman^12^.

### Laser application 

 In this study, the use of low-power laser (LBI) was carried out with the use of a thermocouple (IN-Ga-Al-P) THERAPY^®^ XT^®^ (DMC^®^, Sao Carlos-SP, Brazil) nm ± 10 nm. Eight applications were performed on consecutive days from the 1st to the 8th day of cell culture, with power of 200mW / cm^2^, energy density of 2000mJ / cm^2^, energy dose of 2J / cm^2^, beam area of 0.02mm, irradiation time of 5seconds and application through the culture bottle at only one point of application in the region center of the board.

 Non-irradiated cells were used as controls. Subcultures were maintained for periods up to 18 days under the same conditions described above and their development evaluated under inverted-phase microscopy.

###  Determination of number of cells by tripan blue exclusion method 

 The growth curve was determined by counting in the Neubauer Camera the total number of cells with 1, 8 and 18 days. For cell counting, tissue formed on the surfaces of culture dishes was removed by a solution containing 1.5 ml of 1 mM EDTA, 1.3 mg / ml collagenase (Invitrogen Corporation) and 0.25 trypsin (Cultilab) % in PBS at pH 7.4. An aliquot of the cell suspension was collected and mixed in equal volume of the 1% trypan blue dye. Approximately 20μl were pipetted in each of the chambers and counted under a light microscope with x40 objective, determining the total number of viable cells in the nine reticles of each chamber, according to the protocol described by Freshney^13^.

###  Determination of cell viability by the MTT method 

 After completing the 1, 8 and 18 day periods, the plates were submitted to the cell viability test by the MTT-Formazan colorimetric method. Quantities of 50μl of MTT substrate, diluted in bidistilled water at 5mg / ml concentration, were added to wells protected from light. The plates were then transferred to the CO_2_ oven where they remained for 4 hours. After this time, the plates were brought to the laminar flow; each well received 500μl of SDS and the cell viability determination was performed by measuring the absorbance in an ELISA reader at 570nm, according to Mosmann, T^14^ methodology.

###  Formation of mineralized matrix 

 The alizarin red dye (Sigma-Aldrich) was used to stain the mineralized matrix. The plates were washed with PBS and the cells fixed with 10% formalin solution for 24 hours and, after that period, dehydrated in increasing concentrations of alcohol (30, 50, 70, 90, 96%), each solution being held in contact with cells for 1 hour. After the last hour, the solution was removed and the plates held semi-open until complete drying. They were then filled with a solution of 2% alizarin red pH 4.2 for 8 minutes. Excess dye was removed by plentiful washing of the material with double-distilled water and the plates were again held semi-open until drying. The quantification of the staining was evaluated by colorimetric method according to Gregory *et al.* ^15^.

###  Total protein dosage 

 After 24 hours, 8 and 18 days of plating, the plates were washed three times with slightly warmed PBS and 2 ml of 0.1% lauryl sulfate (Sigma-Aldrich) was placed in each plate, left at room temperature for 30 minutes. 1 ml aliquots of this solution were withdrawn and placed in test tubes along with 500 μl of the Lowry’s solution (Sigma-Aldrich) for 20 minutes at room temperature. 250 μl Folin reagent (Sigma-Aldrich) was added, waiting 30 minutes to read through the spectrophotometer at 600nm. The total protein content was calculated in concentration.

###  Dosage of alkaline phosphatase 

 Twenty four hours, 8 and 18 days after plating, the alkaline phosphatase activity was measured. 1 ml aliquots of the same solution removed from the plates were used to total protein dosage. These aliquots were added to the contents of the commercial kit (Labtest Lagoa Serena) according to the instructions of the kit. The absorbance was calculated by a spectrophotometer at 590 nm and the activity of alkaline phosphatase through a standard curve.

###  Gene expression 

 The evaluation of the gene expression was done through the polymerase chain reaction in real time (real time PCR). The evaluated genes were grouped as follows:

 a) markers of the osteoblastic phenotype: ALP, collagen type 1 (COL I), osteocalcin (OC), OPN. The primers were designed using the Primer Express 3.0 program (Applied Byosystem) (Table 1).

**Table 1:** Primer sequences used for real-time PCR.

Genes	Sequence primer sense	Sequence primer antisense	TA(°C)	TD (°C)	Bp
Osteopontin	AGACACATATGATGGCCGAGG	GGCCTTGTATGCACCATTCAA	58	79	154
Osteocalcin	CAAAGGTGCAGCCTTTGTGTC	TCACAGTCCGGATTGAGCTCA	62	85	150
Type I Collagen	TGACGAGACCAAGAACTG	CCATCCAAACCACTGAAACC	61	84	114
Alkaline phosphatase	ACGTGGCTAAGAATGTCATC	CTGGTAGGCGATGTCCTTA	60	86	475

ALP-alkaline phosphatase, COL I-collagen type 1, OC-osteocalcin, OP-osteopontin; TA - annealing temperature; TD - dissociation temperature; bp - number of base pairs of the product.

###  Evaluation of immunostimulatory properties 

 100 μl of the cell culture medium was withdrawn. 100 μl of the Griess reagent was added and allowed to stand for ten minutes at room temperature, whereupon the concentration of nitric oxide was then determined using the ELISA reader with a 540 nm filter. The results were expressed in micromols (μmols) from a standard curve established in each assay, consisting of known micromolar concentrations of nitric oxide.

###  Cytochemical analysis 

 Xylidine Ponceau (XP) was used for cytochemical analysis to show the total proteins and Toluidine Blue (AT) for DNA, RNA and glycosaminoglycans (GAGS).

###  Oxidative stress through the MDA reaction with TBAR 

 Reactive Oxygen Species (ROS) were determined using the TBARS method^17^ ^,^ ^18^ with some modifications. An aliquot (100 μl) of the sample was added to 1 ml of solution containing 400 μl of 1.3 M acetic acid / 0.27 M HCl buffer, pH 3.4, 400 μl TAB 0.8% and 200 μl SDS 8.1%. The mixture was incubated at 95°C for 60 minutes. The MDA reaction with TBAR produces a chromophore that can be measured photometrically at 532nm. It was evaluated the oxidative stress that occurs during the metabolic process of the culture and intervention, of the laser during the process of cell growth of the Control Group (18 days) and treated with laser (18 days).

###  Statistical analysis 

 All variables were tested for normal distribution and homogeneous variation. When the distribution was considered normal and with the homogeneous variation Student’s t test was performed. In cases where there was no normal distribution, the Mann-Whitney test was used. Differences were considered significant when p <0.05. Statistical analysis was performed using graphPad Prism version 3.0 software.

## Results

 LBI improved cell proliferation when cultures were analyzed by the trypan blue exclusion method.

**Figure 1:**
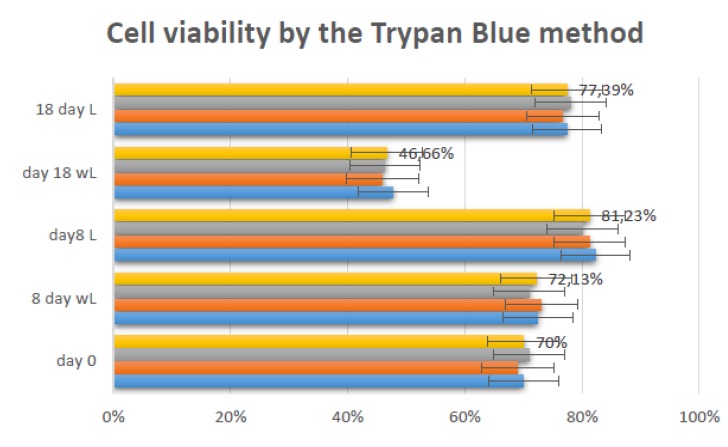
Cell viability by the Trypan Blue method.

 Cells in culture were seeded at a density of 2x10^4^ cells / cm^2^. After irradiation, the number of cells obtained for group A at 8 days of culture were 1.36x105 cells / cm^2^ with 82.3% of viable cells. Group B had a mean viability of 72.4%. At 18 days, Group A presented cell viability of 1.55x10^5^ cells / cm^2^ with 77.42% of viable cells. In Group B, cell viability was 1.05 x 10^5^ cells / cm^2^ with 47.62%. Figure 1 illustrates viable and non-viable cells in the presence of trypan blue dye. Figure 1 represents the cellular reliability in triplicate, expressing the values of the means at day zero, after 8 days of the control, 8 days with laser, 18 days of the control and 18 days with laser.

 Evaluation of cell proliferation at different times by MTT indicated an increase in cell numbers between 8 and 18 days. The mitochondrial activity measured by absorbance in 24 hours of culture was 47.7%. At 8 days, the culture without laser application obtained viability of 65.5%; already with laser, in the same period, viability was 80.4%. In cultures of 18 days not submitted to laser, the viability was 41.2% and 79% in those cultures irradiated in the same period of 18 days.

**Figure 2:**
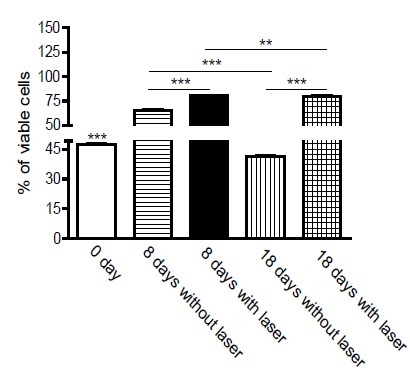
Determination of viability by MTT test.

 In Figure 2, the viability of the osteoblast culture was analyzed 24 hours after mechanical digestion of the bone explant and without laser intervention, culture of 8 and 18 days with and without laser treatment.

 When we observe the comparative analysis through the T test, P*** values are significantly different, p <0.0001.

**Figure 3:**
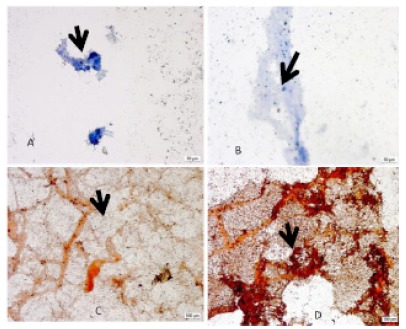
Details of culture of osteoblasts with 18 days. Cultures **A** (x20) and **C** (x10) without laser. Cultures **B** (x10) and **D** (x20), irradiated with laser. The arrow identifies the nuclei.

 Dye Xylidine Ponceau (XP) showed the total proteins and blue of toluidine (AT), DNA, RNA and glycosaminoglycans (GAGS). It can be seen in Figure 3 that, in TA staining, the laser irradiated groups present the most stained nuclei in relation to the cytoplasm (B). The nucleoli were large and well defined when compared to the non-irradiated group. The number of cells per field observed under an optical microscope evidenced a higher number of cells in the group irradiated with laser. In samples stained by XP, it can be observed that the cytoplasm presented with hyperchromasia. The total proteins stained in more intense red made it very evident in the group irradiated with laser that its presence occurred in greater quantity5 (D).

 Total protein dosage expresses the increase of collagen and non-collagen proteins. The total protein measure was affected by laser treatment. The results obtained at the end of 8 and 18 days with laser were significantly higher when comparing the times and the groups in Table 2. The concentration averages of 16.604 mg/mol were obtained in the control group; 21.622 mg/mol, in the 8-day laser irradiated group; 24.95 mg/mol, control group at 18 days and 37.25 mg/mol laser irradiated group at 18 days.

**Table 2:** Total protein (μg/10^4^ cells) of 8 and 18 days of culture. Irradiated or not with laser.

Control		Laser	
8 days	18 days	8 days	18 days
15.02	20.16	23.7	32.28
16.11	21.7	26.61	39.92
17.12	22.25	25.19	41.3
18.42	22.17	26.08	37.65
16.35	21.83	23.17	35.1

**Figure 4:**
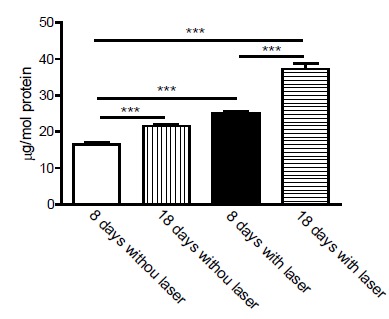
Total proteins per mg/mol.

 In Figure 4, we can observe that: a ≠ C8 for p = 0.00007; b ≠ L8 for p = 0.0001; c ≠ C8 when p = 0.00005; d ≠ C18, p = 0.00001. When total protein values were submitted ANOWAY ANOVA for two factors of variation and comparison between treatment type and time, we observed that the amount of total protein was statistically significant in relation to all groups compared each other.

**Figure 5:**
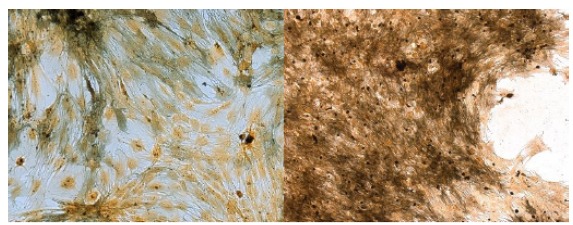
Histochemical reaction for the presence of alkaline phosphatase. Cell cultures performed in different experimental conditions, macroscopic aspect, where in **A** it is observed culture of osteoblasts without the presence of laser. In **B**, the laser irradiated osteoblast culture (Light Microscopy x10).

 Cell cultures were stained for the presence of alkaline phosphatase (Fig. 5). The yellow, brown or black coloration is identified as a positive reaction. The darker color is evidenced by the larger amount of the enzyme alkaline phosphatase. The areas stained with black alkaline phosphatase were measured to verify the result^9^.

###  Formation of mineralized matrix 

 The formation of mineralized matrix expresses the increase of the formation of areas of calcification. This matrix was expressed as absorbance and calcium was evidenced through the alizarin red dye. The presence of the mineralized matrix is visualized by red dots, which indicate the presence of calcium in the cells of the control and laser irradiated groups.

**Figure 6:**
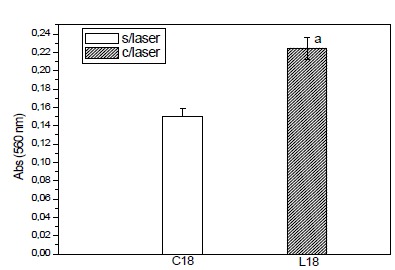
Mineralized matrix. Relationship between irradiated (laser) and non-irradiated (laser) groups. Absorbance units (UA).

 The evaluation of these parameters was performed by biochemical methods and observation of the cultures by light microscopy using a methodology established by Coelho and Fernandes^9^. The values obtained in absorbance through the spectrophotometer for the mineralized matrix are described in Table 3 and Figure 6. The values were obtained in the control group 18 days and group irradiated in 18 days. Samples were obtained in quadruplicates, for control group and group irradiated with laser 18 days. 

**Table 3:** Absorbance values for the mineralized matrix.

**Control**	0.152	0.139	0.16	0.149
**Laser**	0.21	0.219	0.234	0.235

P=0.00005 Anova

 Positive results, obtained through RT-PCR reactions for OPN, OCN, ALP and COL I, were able to confirm, in established cultures, the expression of the proteins present in the osteoblast differentiation and maturation stages.

 The data obtained in this analysis were analyzed using the One Way ANOVA test with values of p = 0.00005 with significant differences between the culture groups with 18 day incubation time for the control groups (without laser) and irradiated group.

**Figure 7:**
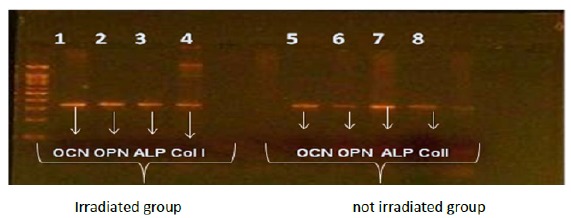
Analysis of 2% agarose gel PCR products in the osteoblastic cultures submitted to 18 days (bands 1-4) and control (bands 5-8).

 In Figure 7, the bands for the OCN, OPN, ALP and Col-I genes were intact in the cell group submitted to laser exposure, as well as to the control group.

 Evaluation of immunostimulatory properties. Determination of Nitrite (NO, TNFalpha IL6)

**Figure 8:**
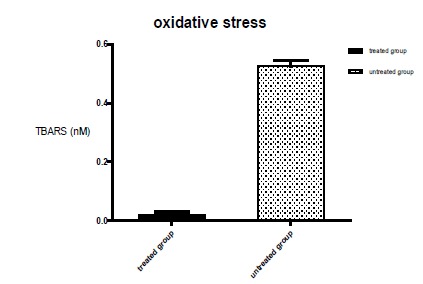
Final products of lipoperoxidation in the soluble fraction of the culture medium of the treated and untreated groups. After the data collection, the results had values of P<0.05 were considered statistically significant. P = 0.0001 when compared to the reference group. (Unpaired Student’s t-test, ANOVA variance analysis).

 The results demonstrated a significant decrease in the final product levels in the Experimental Group when compared to the Control Group standard curve (Fig. 8).

## Discussion

 In this work, the effect of GaAlAs laser irradiation on osteoblast culture from femur surgical explant of adult male Wistar rats was evaluated. The purpose of the study was to analyze the behavior of osteoblasts by low-power laser performance in a controlled environment.4, seeking to contribute to the knowledge of the mechanisms that act on bone cell units in the presence of stimulant light. The dose of 2 joules was selected through studies described in the literature researched in in vitro models^4^ ^,^ ^5^ ^,^ ^10^. The time periods of culture and exposure to laser light were selected because they represent periods of intense cellular activity^5^ ^,^ ^25^. By specific parameters, the laser can increase cell proliferation. Some researchers have stated that different results are cumulative. Other authors have reported that laser light interferes with the hydrogen acceptors of the respiratory chain, promoting the transmission of the signal that can be translated intracellularly as proliferation and gene expression^25^ ^,^ ^26^. The experimental conditions used in the primary culture, culture of cell suspension obtained through the mechanical and proteolytic digestion of bone femoral explant of α-MEM mice, fetal bovine serum, ascorbic acid, antifungal and antibiotic, allowed to isolate a population of osteoblastic cells^9^ ^,^ ^10^ ^,^ ^16^. The additives and reagents used followed careful bibliographical analysis in experimental models with the same characteristics^9^. In order to develop the osteoblast culture, the addition of ascorbic acid (AA), an important inducing agent in the production of collagenous extracellular matrix^9^ is necessary^9^. The collagenous matrix is a fundamental matrix for the expression of the osteoblasto phenotype^25^. This collagenous matrix gains a rigid structure, derived from the betaglycerophosphate added to the culture to provide phosphate which is subsequently hydrolyzed by alkaline phosphatase to proceed to matrix mineralization^26^ ^,^ ^27^. As an inducing osteoporous substance in cell culture, dexametazone is routinely used to promote expression of phenotypic parameters such as: alkaline phosphatase, osteopontin, osteocalcin and increased extracellular matrix mineralization^10^. The cells used in this work, derived from the bone explant of the femur of rats, were confirmed by tripan blue staining and MTT reaction, compatible with other experiments^14^. They proliferated and developed the osteoblastic phenotype, as they presented positive activity for alkaline phosphatase. Alkaline phosphatase (ALP) is one of the biomarkers used to evaluate bone metabolism; Coloring methods are also used to visualize the formation of bone nodules^9^. However, when the results presented by the group irradiated by the laser were observed, the differences were significant in relation to the cellular behavior related to growth and cell viability. This observed data set suggests that laser irradiation affects osteoblastic cells^28^.

 Success in culturing any type of cell can only be achieved if there is a specific phenotype marker, allowing confirmation of cell identity in vitro. In this work, we try to evidence the presence of osteocalcin, osteopontin and osteonectin as phenotypic markers. In terms of osteoblastic cultures, osteocalcin is undoubtedly considered the most specific marker for osteoblastic phenotype^29^; therefore, the expression of this protein has been evidenced in the cultured cells. Other authors also used markers for OPN, ALP, OCN and COLI^4^ ^,^ ^10^. The results found in this study were similar to those of these authors. Alizarin red staining has been used to verify the ability of cells to produce mineralization nodules and our results corroborate the results obtained in these studies, in which osteoblastic cells obtained from the cultures also determined the formation of in vitro mineralization nodules^30^. Free radicals are involved in bone metabolism. Increased hydrogen peroxide may elevate the expression of TNFα that has the ability to induce osteoclastogenesis. Hydrogen peroxide itself elevates the metabolism and facilitates the differentiation of osteoclasts, which produce large numbers of reactive oxygen species that in smaller amounts stimulate cell growth. This intense oxidative stress increases the damage in cellular DNA, inducing apoptosis^22^. This evidence suggests that laser radiation stimulates the formation of calcified matrix. The increase of hydrogen peroxide may increase the expression of TNFα^23^. The reactive oxygen species, in recent studies, are identified as the main secondary messengers produced by low intensity leisure light (LLLT)^19^ ^,^ ^20^. For some authors, laser therapy influences parameters of oxidative stress, such as alteration of the activity of antioxidant enzymes and the production of reactive oxygen species (ROS). Electron transfer increases the production of superoxide anion and triggers an initial production of ROS. As already described by Assis^21^, the excess production of these species can lead to damages in the cellular constituents. Depending on the dose, exposure time and intensity, low intensity laser radiation (LLLT) modulates biochemical processes in the increase in the antioxidant system related to decreased tissue damage, increased mitochondrial respiration and ATP synthesis^23^.

 The results of the present study showed that the irradiation of low intensity infrared laser light 808nm (In-Ga-Al-P) at various energy levels affected the physiological and molecular properties of cells, interfering with the proliferative activity. In addition, our results indicate that cells can grow effectively during laser irradiation, in vitro.

 The markers of the RT-PCR reactions found in the 18 day laser cultures were very similar to those in the control group. According to Garland^3^, being considered as proof of the phenotypic characteristics of cells of osteoblastic cultures.

## Conclusions

 As can be observed, the application of laser radiation with 808nm, energy dose of 2J/cm^2^, irradiation time of 5s in culture plates; allows to conclude:

 The irradiated cultures presented higher cell viability, matrix mineralization and maintained the cellular expression of the proteins. The final products of the lipoperoxidation in the soluble fraction of the culture medium of the treated groups were expressively lower in the irradiated group. The experimental model used was adequate for this type of observation.
